# Designing a meditation module to affect etiological and preventive factors in primary hypertensive patients—A pilot study

**DOI:** 10.14814/phy2.70226

**Published:** 2025-02-06

**Authors:** Kapila Goswami Sharma, S. D. Manjula, Shobha U. Kamath, M. Mukhyaprana Prabhu, M. G. Ramesh Babu, Ujjal Bose, K. Vasanthalaxmi

**Affiliations:** ^1^ Department of Physiology Kasturba Medical College, Manipal, Manipal Academy of Higher Education (MAHE) Manipal Karnataka India; ^2^ Department of Biochemistry Kasturba Medical College Manipal, MAHE Manipal Karnataka India; ^3^ Department of Medicine Kasturba Medical College Manipal, MAHE Manipal Karnataka India; ^4^ Division of Physiology, Department of Basic Medical Sciences MAHE Manipal Karnataka India; ^5^ Department of Pharmacology American University of Antigua College of Medicine Osbourn Antigua and Barbuda

**Keywords:** brain‐derived neurotrophic factor, complementary and alternative therapy, essential hypertension, meditation based intervention, primary hypertensive patients

## Abstract

The aim of the present pilot study is to design, validate, and check the feasibility and efficacy of the designed meditation module on etiological and preventive factors in primary hypertensive patients (PHP). The systematic and detailed meditation module was formulated to prevent the complications of hypertension. The designed module was validated by 5 subject experts in the field of complementary and alternative therapy and research. Content Validity Ratio (CVR), Average Congruency Percentage (ACP), and Content Validity Index were calculated. The intervention was given for 5 days a week for 4 weeks, to 9 PHP to check the feasibility and the efficacy of the module by analyzing biochemical and psychological parameters. A randomized controlled trial is planned ahead with this module as intervention. The designed meditation module had 19 concepts with CVR >0.49 and ACP > 90% which were retained, 2 were modified (CVR 0.6, ACP 80) and 1 was deleted as CVR was <0.49 and ACP < 70%. The feasibility test mean was 93.33 ± 8.66 and the efficacy was tested by increase in the brain‐derived neurotrophic factor (BDNF) (*p* = 0.022) and decrease in anxiety (*p* = 0.022) and perceived stress (*p* = 0.016), improvement in emotional quotient (*p* = 0.011), mental state (*p* = 0.044), quality of sleep (*p* = 0.001), quality of life (*p* = 0.011), and happiness state (*p* = 0.003). The designed meditation module was found valid by the experts, feasible to patients, and efficient in improving biochemical and psychological parameters among PHP to affect the hypertensive etiological factors and check the progress and complications of the disorder.

**Trial Registration:** CTRI/2022/09/045421

## 1 INTRODUCTION

Hypertension is affecting 1.28 billion people worldwide between the range of 30–79 years of age and is one of the global targets for non‐communicable diseases. It is also among one of the major causes for premature death especially in low‐ and middle‐income group countries (World Health Organization, 2023). The number of people being affected by hypertension is predicted to increase by 60% in 2025 to 1.56 billion as compared to 1 billion in 2000 (Kearney et al., 2005). The prevalence of hypertension around the world and its complications are a concern especially in developing country like India and thus arises need of methods to prevent and control it. The non‐pharmacological methods, for the prevention and control of hypertension are in due to no or minimal side effects on health, as dietary modifications, lifestyle modifications, acupuncture, yogic relaxation techniques, mindfulness and meditation‐based interventions (Verma et al., 2021). The meditation and mindfulness‐based interventions show potential lowering effects on blood pressure levels (Geiger et al., 2023). The mechanism responsible for it involves reduction in sympathetic activity along with slow and deep breathing during the process. Slow breathing results in low‐frequency oscillations in blood pressure and synchronization of heart rate at the low respiratory frequency, thus inducing chemoreflex sensitivity reduction and arterial baroreflex sensitivity increase which ultimately leads to parasympathetic dominance (Bernardi et al., 1998). The self‐ regulation of thoughts, control of attention bringing change in anterior cingulate cortex region, regulation of emotions involving fronto‐limbic networks, awareness about the self and present moment altering midline prefrontal cortex and posterior region of cingulate cortex, and reduction of stress are the concepts involved in the process of meditation (Tang et al., 2015). A systematic review and meta‐analysis, on blood pressure response to meditation shows that in some cases meditation is safe and effective alternative to pharmacotherapy (Park & Han, 2017). Yoga as Antihypertensive Lifestyle Therapy: A Systematic Review and Meta‐analysis concluded that yoga places greater benefits when meditation is included as part of therapy (Wu et al., 2019). Different types of meditation are practiced along the globe with different concept and effect so the need to formulate a module which starts from mindfulness and moves to meditation especially to control symptoms and complications of primary hypertension was felt.

As there is decrease in the brain derived neurotrophic factor (BDNF) levels in chronic diseases such as hypertension, so assessment of BNDF might establish correlation between the two (Hang et al., 2021). There is also evidence of increase in BDNF with the help of meditation (Gomutbutra Patama et al., 2020). Evidence of the role of angiotensin II in the regulation of blood pressure exists (Fyhrquist et al., 1995), and its level when remains within the range might help in the control of high blood pressure so efficiency of a module also depends on the way it regulates the level of angiotensin II. The level of cortisol is associated with normal high blood pressure thus facilitating development of hypertension (Erin et al., 2021), a module which might decrease the cortisol level might help in the development of the disease. Correlation between lipid profile and the development of hypertension proves (Chruściel et al., 2019) that the risk factors for the cardiovascular diseases can be decreased by changes in the lipid profile. Non‐adherence to intervention due to less feasibility, high complexity, and time constrains might diminish the formulation of any intervention‐based trial so the success of a developed module also depends on the feasibility and high adherence.

As stress and anxiety are the etiological factor for the development of hypertension (Kulkarni et al., 1998) so a module to decrease stress and anxiety would help to control the progress of the disorder. Emotional regulation is often challenged in hypertensive patients and through meditation patients are able to regulate their emotions better and frequency of the negative emotions also decrease which overpowers the positive emotions (Basso et al., 2019). Correlation of subjective sleep quality and hypertension (Lo et al., 2018) is found and meditation relaxes mind and body to induce sleep and decrease disturbances thus influencing overall sleep quality with respect to duration, latency, efficiency, disturbances daytime dysfunction, and use of sleep medication. Most of the hypertensive patients report poor or disturbed mental state due to association of mental health, blood pressure, and hypertension (Schaare et al., 2023) and meditation changes the thought process and behavior thus improving the overall mental health. Emotional arousal and anger are associated with changes in blood pressure and are contrary to the experience of happiness (James et al., 1986), meditation is found to change the baseline measures of happiness and well‐being by increasing sense of compassion and detachment. Hypertension affects all the domains of the quality of life of the patients and there is difference in the quality of life of meditators and non‐meditators. The aim of the present study is to design and validate a module for primary hypertensive patients based on classical and scientific literature of complementary and alternative therapies and test its feasibility and efficacy by giving intervention to patients and analyzing biochemical and psychological parameters to understand how it is affecting etiological factors of hypertension and acts as a preventive measure to develop complications of hypertension.

## 2 MATERIALS AND METHODS

After the detailed analysis of etiological and preventive factors of hypertension, this study was planned in 2 stages:

- Stage 1: Designing of the meditation module for the primary hypertensive patients
- Stage 2: Validating the designed module by the subject matter experts in the field of complementary and alternative therapy and feasibility and efficacy testing of the designed meditation module.

### 2.1 Stage 1: designing of the meditation module for the primary hypertensive patients

It comprised designing of meditation module specifically for Primary Hypertensive Patients (PHP) from the classical texts of complementary and alternative philosophy and therapy and scientific text on physiology of meditation practices. Module was designed keeping in mind the etiological factors of hypertension and complications of the disorder based on modern medicine with solution from prospective of complementary and alternative therapy. Classical texts which were referred were Patanjali Yoga Sutras (Taimni, 1992), Siva Samhita (Vasu, 2012), Hathayoga Pradipika (Svatmarama, 1994), Gheranda Samhita (Digambarji & Gharote, 1978), Vedantsara (Yogindra, 1978), and Samkhya Karikas (Krishna, 1872). The philosophical texts referred were Bhagavad Gita (Ved, 1972) and Srimad Bhagavatam Mahapurana (Vyasa, 2000). The scientific text referred was Anatomy and Physiology of Yogic Practices (Gore, 2017). Modern medicine text referred for etiology, complication and pathophysiology of hypertension was Medical Physiology (Guyton and Hall, 2020). Research papers were reviewed for the types of meditation and concept behind them and also for the management of hypertension through meditation. Designed Meditation Module (DMM) was formulated keeping in mind that it would help in controlling blood pressure, controlling symptoms and complications of hypertension, improve quality of life and sleep, emotional quotient, mental state, happiness scale, and decrease stress and anxiety among PHP. A module of 48 min (Table 1) was formulated for patients to be practiced 5 days a week for 4 weeks for the pilot study as per the guidelines of Institutional Ethical Committee, Kasturba Medical College, and Kasturba Hospital, Manipal.

**TABLE 1:** Content of designed meditation module based on classical and scientific text on complementary and alternative therapy and literature review.

S.No.	Explanation about the concept	Duration in minutes
1	Sitting in Swastikasana (Auspicious Pose)	1
2	Normal breathing	1
3	Awareness of distant sounds	1
4	Awareness of near sounds	1
5	Awareness of body	1
6	Breathing observation	2
7	Thoughts observation	3
8	Visualization of gross aspects of heart and cardiovascular system	7
9	Visualization of water body	1
10	Visualization of subtle aspects of heart	
	Anahat Charkra (Heart Center) Dhyan	1
	Hridaya Padma (Heart compared to a Lotus) Dhyan	1
	Chanting Yam (Seed sound of Heart Center)	1
	Prana Vayu (Life Force) Sensation	1
	Evoking Priti (Love)	1
	Concentration on flame	1
	Feeling Ananda (Bliss)	1
11	Gross and subtle aspects of body	8
12	Self‐Consciousness Awareness	3
13	Universal Consciousness Awareness	3
14	Merging of self and Universal Consciousness	4
15	State of Salutogenesis (Self‐Healing)	3
16	Reversal to normal state	2

### 2.2 Stage 2: validating the designed module by the subject matter experts in the field of complementary and alternative therapy

This stage started with validation of the designed module by the subject matter experts (SMEs) in the field of complementary and alternative therapy for more than 5 years, who had formal education in the field of meditation with valid post‐graduation or medical degree, teaching meditation as a therapy to patients in a recognized institution or at the center. The exclusion criteria for the experts in the validation work was experts with experience less than 5 years, no formal degree or education and not practicing at the moment. It included experts from varied fields as yoga instructor, yoga therapist, yoga teacher, researcher in the field of yoga, and medical practitioner in yoga as meditation being the part of yoga. A relative search was done for the list of experts in the field of yoga, the name of universities, recognized centers, and school of yoga they are associated with. The experts were short listed based on the inclusion and exclusion criteria (Table 2). The demographic of experts was four Females (80), 1 Male (20) with educational qualification ranging from Medical Director, Selection Grade Lecturer, Assistant Professor‐2, Founder of Yoga Centers. The communication through mail or phone was done to get their concern to participate and then module with instructions was shared to respond. A 5‐point rating scale was given for each concept based on the effect ranging from essential so cannot be eliminated 1, less essential but can be retained 2, neutral effect 3, unnecessary so can be eliminated 4, not required at all 5. A write up was given to experts which had instructions to fill the table containing designed meditation module, briefing about the module and personal details as designation, years of experience in the field of yoga, educational qualification, institution, and additional comments were collected. Data were analyzed for content validity using Lawshe Content Validity Ratio (CVR) method for each concept (Lawshe, 1975). Average Congruency Percentage (ACP) (Popham, 1978) and Content Validity Index (CVI) (Martuza, 1977) was also calculated for the module. If the score of 1, 2 and 3 was given then it was considered 1 to retain the concept but if 4 and 5 score was given then it was considered 0 either to remove or modify the concept. The minimum value of 0.49 CVR and ACP of 70% was required in order to be statistically reliable. Modifications in the module were based on the response of the experts if the content validity of the concept was found to be less than 0.49 CVR and ACP was less than 70%. Designed meditation module was also checked for the quality based on Yoga Module Development and Validation Methodological Guidelines (Katla et al., 2022). The score of the module was 19 out of 23 which falls under high quality (Table 3).

**TABLE 2:** Flowchart of expert assigned to validate the designed meditation module.

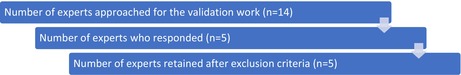

**TABLE 3:** Details of the 23‐item checklist for the quality of the module based on Yoga Module Development and Validation Methodological Guidelines.

Domain	Item number & checklist items	Reported: Yes or No
Yoga module development	1 Traditional literature review	Yes
	2 Scientific literature review	Yes
	3 Dose, frequency, and duration of intervention	Yes
	4 Involvement of experts, clinicians and participants in the development Phase	Yes
	5 Yoga practice sequence	Yes
	6 Customization of practices	Yes
	7 Instructor qualification	Yes
	8 Reporting adverse effect	Yes
	9 Home practice details	Yes
Yoga module validation	10 Professional eligibility of experts	Yes
	11 Diversity of experts	Yes
	12 Details of the items provided to experts	Yes
	13 Involvement of stakeholders	No
	14 Expert content validation	Yes
	15 Group discussion	No
	16 Use of case vignettes	No
	17 Statistical procedure for item retention	Yes
	18 Involvement of stakeholders	No
	19 Modifications made after validation	Yes
Yoga module feasibility	20 Feasibility phase done	Yes
	21 Measurement of intervention fidelity	Yes
	22 Feedback from participants	Yes
	23 Outcome domain	Yes

### 2.3 Feasibility and efficacy testing of the designed meditation module

The DMM was given to small group of primary hypertensive patients, who were diagnosed as primary hypertensive by the Medical Doctor, to test its feasibility and efficacy. A pilot work was conducted by recruiting patients from OPD of TMA Pai Hospital, Udupi, Karnataka, India, for the period of 6 months, after taking ethical clearance from the Institutional Ethical Committee, Kasturba Medical College and Kasturba Hospital, Manipal (IEC 1‐154) and CTRI Registration (CTRI/2022/09/045421). The study was performed in accordance with the ethical standards as laid down in the 1964 Declaration of Helsinki. Prior to recruitment of patients, permission from the Head of the Institution and Medical Superintendent, TMA Pai Hospital to recruit the patients from OPD and signature on consent form and participation information sheet from the patients to participate in the study were taken. The permission to use the questionnaires was taken by respective authors. Consented patients were communicated through the phone and messages about the details of the venue, time and pre‐requisite of meditation as they should take bath before coming to session, should not eat anything, wear loose clothes and bring mat to sit.

Inclusion criteria for patients to participate in the study was Primary Hypertensive Patients with or without medication of both genders, age between 18 to 60 years, Systolic Blood Pressure less than 180, Diastolic Blood Pressure less than 120 mm of Hg and Exclusion criteria was Patient with Secondary Hypertension, mental disorder, pregnant ladies, meditation practitioners, patients with cancer.

The sample size of 9 (6 females, 3 males) was approached after screening 35 patients (Table 4). The average age of the patients was 51 years ± 8.1 years. The socio‐demographic data and body parameters of the patients is given in Table 5. Intervention was given for 4 weeks at TMA Pai Hall, Udupi following the IEC guidelines. A briefing about the study and the intervention was given to all the patients who consented to be part of the study. As per the guidelines of IEC, the intervention was given to patients for 4 weeks, 5 days a week excluding Saturday and Sunday for 48 min by gradually increasing from 15 to 48 min in the 1st week and for rest of the 3 weeks it was maintained as 48 min. 1st week and 4th week intervention was given onsite between 7 to 8 in the morning by the therapist and 2nd and 3rd week a recorded audio session of the same was given to the patients to practice (Table 6). A diary was given to mark the days intervention was practiced by the patients and a call was made to all patients to enquire if they were doing the session or had any complications. 5 mL blood was withdrawn from the patients at the laboratory of TMA Pai Hospital, Udupi by the lab technician before starting the intervention and the questionnaires were given to them to fill along with data collection sheet. Separation of serum was done in the laboratory immediately after collection and serum was stored at −80^0^ Centigrade for analysis. After 4 weeks post‐test was done for which again 5 mL blood was collected and questionnaires were given. Traveling and participating allowance was given to patients as per the IEC guidelines.

**TABLE 4:** Flowchart of recruitment of patients for the study.

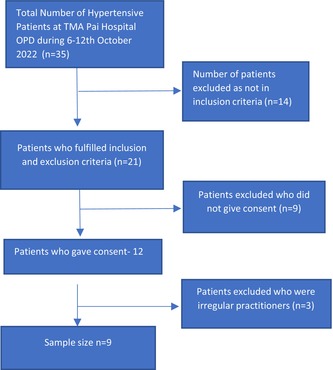

**TABLE 5:** Socio‐demographic data and body parameters.

Socio‐demographic and body parameters details	Mean	Standard deviation
Socio‐demographic details		
Age (in years)	51	8.10
Gender *n* = 9 (%)	Male‐3 (33) Female‐6 (67)	
Profession	Retired‐1 (11) Home maker‐5 (56) Working‐3 (33)	
Family members	Three‐3 (33.33) Four‐3 (33.33) Five‐1 (11.11) Six‐2 (22.22)	
Food habits	Vegetarian‐5 (56) Non‐vegetarian‐4 (44)	
Type of treatment	Allopathic‐8 (89) Allopathic+Ayurvedic‐1 (11)	
Family history	BP to Father+Mother‐3 (33) BP to Mother‐ 6 (67)	
Duration (in years)	5.55	2.18
Co‐morbidities	None‐7 (78) BA‐1 (11) OB + LBA‐1 (11)	
Body parameters		
SBP/DBP (millimeters of mercury)	131.33 85.55556	15.03 8.69
BMI (kilograms per square meter)	24.41	3.81
Pulse (beats per minute)	76.88	7.76
Respiratory rate (breaths per minute)	15.88	3.78
Bowels	Regular‐6 (67) Irregular‐3 (33)	

Abbreviations: BA, Mild Bronchial Asthma; OB, Obesity; LBA, Low Back Ache.

**TABLE 6:** Explanation of days on which intervention was given to the patients along with the details of the mode and pre‐ and post‐tests days.

Date and day	Detail about Intervention	Week	Date and day	Detail about intervention	Week
28th Oct, 2022, Fri	Pre‐test, Onsite	1st	10th Nov, 2022, Thur	Gap	2nd
29th Oct, 2022 Sat	Onsite	1st	11th Nov, 2022, Fri	Audio	3rd
30th Oct, 2022, Sun	Onsite	1st	12th Nov, 2022, Sat	Audio	3rd
31st Oct, 2022, Mon	Onsite	1st	13th Nov, 2022, Sun	Audio	3rd
1st Nov, 2022, Tue	Audio	1st	14th Nov, 2022, Mon	Audio	3rd
2nd Nov, 2022, Wed	Gap	1st	15th Nov, 2022, Tue	Audio	3rd
3rd Nov, 2022, Thur	Gap	1st	16th Nov, 2022, Wed	Gap	3rd
4th Nov, 2022, Fri	Audio	2nd	17th Nov, 2022, Thur	Gap	3rd
5th Nov, 2022, Sat	Audio	2nd	18th Nov, 2022, Fri	Onsite	4th
6th Nov, 2022, Sun	Audio	2nd	19th Nov, 2022, Sat	Onsite	4th
7th Nov, 2022, Mon	Audio	2nd	20th Nov, 2022, Sun	Audio	4th
8th Nov, 2022, Tue	Audio	2nd	21st Nov, 2022, Mon	Onsite	4th
9th Nov, 2022, Wed	Gap	2nd	22nd Nov, 2022, Tue 23rd & 24th Nov 2022, Wed &Thur	Onsite, Post‐test	4th
				Gap	

*Note*: Light Brown Box‐ Pre‐test/Post‐test along with onsite intervention, Yellow Box‐Onsite intervention, Red Box‐Supposed to be onsite but audio session was given, Lavender‐ Gap day, Light purple‐Audio session.

The feasibility testing was done for convenience in performing the entire module, percentage of patients completing sessions for 1 month and for number of days, problems faced during the intervention period. Overall feedback from the patients was taken about the session. The efficacy of the module was tested by difference in the values of brain‐derived neurotrophic factor, Angiotensin II, Cortisol, lipid profile as mark of biochemical parameters and scores of reliable and valid questionnaires, after permission to use, for Emotional Quotient (Multi‐Dimensional Emotional Question), Mental State (Mini Mental State Exam), Quality of Life (World Health Organization Quality of Life – BREF) and Sleep (Pittsburgh Sleep Quality Index), Happiness score (Oxford Happiness Questionnaire), Stress (Perceived Stress Scale) and Anxiety (Hamilton Rating Scale for Anxiety) as psychological parameters before and after the intervention. The laboratory of Department of Basic Medical Sciences (formerly known as Melaka Manipal Medical College), Manipal Academy of Higher Education, Manipal was used to perform analysis of biochemical parameters BDNF (E‐EL‐H0010), Angiotensin II (E‐EL‐H0326) and cortisol (E‐EL‐0157) through ELISA kit as per the instructions given in the kit and microplate ELISA Reader was used to note the optical density values before and after the intervention. Lipid profile was done at the laboratory of TMA Pai Hospital, Udupi by the laboratory technicians.

All the patients completed the session for the entire intervention period. The minor complains faced by the 1 or 2 patients in the first week of intervention were slight heaviness in head, constipation, and pain in back due to sitting for long in one posture which subsided in the 2nd week and after. Apart from this no adverse effect of the intervention was reported by the patients.

## 3 RESULTS

Based on the classical and scientific literature a module with 22 concepts (16 main and 10th having 7 sub‐ concepts making total of 22) was designed which was validated by 5 SMEs among which 4 were females and 1 male with average experience in the field of yoga therapy 19.8 ± 13.42 years. The content validity ratio was calculated by Lawshe formula (Table 7). The concepts with value >0.49 were retained and concepts with value <0.49 were either modified/deleted. Among 22 concepts, 19 were retained, and 2 were deleted, and 1 was modified. Average congruency percentage (ACP) was also calculated for the module, and the value >70% was considered to be valid. The mean ACP of the module was 95.45 (Table 7). Content Validity Index for individual items (I‐CVI) was calculated, and mean I‐CVI was 0.95. The Content Validity Index for the overall scale was calculated based on Average (S‐CVI Average or S‐CVI/Ave) and Universal Agreement (S‐CVI Universal Agreement or S‐CVI/UA). S‐CVI/Ave value was 0.924, and the S‐CVI/UA value was 0.95.

**TABLE 7:** Content validity ratio (CVR), Average congruency percentage (ACP), and Content Validity Index (CVI) of the Designed Meditation Module.

S.No	Explanation about the concept	Expert 1	Expert 2	Expert 3	Expert 4	Expert 5	CVR	ACP	CVI
1	Sitting in Swastikasana	3	1	2	1	2	1	100	1
2	Normal breathing	1	1	1	1	1	1	100	1
3	Awareness of distant sounds	1	4	1	4	1	0.2	60	0.6
4	Awareness of near sounds	1	4	1	4	1	0.2	60	0.6
5	Awareness of body	1	4	1	1	1	0.6	80	0.8
6	Breathing observation	1	1	1	1	1	1	100	1
7	Thoughts observation	1	1	1	1	1	1	100	1
8	Visualization of gross aspects of heart and cardiovascular system	1	1	1	2	2	1	100	1
9	Visualization of water body	1	1	1	2	2	1	100	1
10	Visualization of subtle aspects of heart								
	a. Anahat Charkra Dhyan	1	1	1	1	1	1	100	1
	b. Hridaya Padma Dhyan	1	1	1	1	2	1	100	1
	c. Chanting Yam	1	1	1	1	1	1	100	1
	d. Prana Vayu Sensation	1	1	1	1	2	1	100	1
	e. Evoking Priti	1	1	1	1	2	1	100	1
	f. Concentration on flame	1	1	1	1	2	1	100	1
	g. Feeling Ananda	1	1	1	1	1	1	100	1
11	Gross and subtle aspects of body	1	1	1	1	1	1	100	1
12	Self‐Consciousness Awareness	1	1	1	1	1	1	100	1
13	Universal Consciousness Awareness	1	1	1	1	1	1	100	1
14	Merging of self and Universal Consciousness	1	1	1	1	1	1	100	1
15	State of Salutogenesis (Self‐Healing)	1	1	1	1	1	1	100	1
16	Reversal to normal state	1	1	1	1	1	1	100	1

If score is 1 (Essential so cannot be eliminated), 2 (Less Essential but can be retained), 3 (Neutral Effect) is given by the experts then the concept is retained and if the score is 4 (Unnecessary so can be eliminated), 5 (Not required at all) then the concept is deleted or modified. For the calculation of CVR, score 1, 2, 3 are considered as 1 and score 4, 5 are considered as 0.

For the feasibility testing (Table 8) the mean value was 93.33 ± 8.66 based on the attendance diary, all the patients in the sample size completed the session and had convenience in performing and no major adverse effect was reported.

**TABLE 8:** Feasibility test results of the DMM.

Detail of items	Mean	SD
Attendance (Based number of days session done)	93.33	8.66
Number of patients completed session	In PC 9 out of 12 (75) In SS 9 out of 9 (100)	
Convenience	All patients reported the module was convenient to perform and had soothing effect	
Problem Faced	No major issues observed	

Abbreviations: PC, Patients who gave Consent; SS, Sample Size.

The biochemical parameters such as BDNF, Angiotensin II, cortisol, lipid profile, and questionnaires to assess psychological parameters such as quality of life and sleep, emotional quotient, mental state, happiness, anxiety, and stress were assessed pre and post intervention. The data were analysed by paired *t* test. The mean values of biochemical and psychological parameters pre and post intervention with mean, standard deviation, and *p* value are shown in Table 9.

**TABLE 9:** Data of biochemical and psychological parameters.

	Mean‐pre	SD‐pre	Mean‐post	SD‐post	*p*‐Value
Biochemical Parameters					
1. BDNF (pg/mL)	402.2	446.7	568.1	530.2	0.022
2. Angiotensin II (pg/mL)	5680	3592	5460	3645	0.613
3. Cortisol (ng/mL)	724.3	201.4	618.7	114.6	0.057
4. Lipid Profile					
Serum Cholesterol (milligrams per deciliter (mg/dL))	172.44	26.66	170.66	42.77	0.881
Serum Triglycerides (mg/dL)	109.22	34.19	102.77	14.55	0.527
HDL Cholesterol (mg/dL)	50	6.80	50.11	11.39	0.972
LDL Cholesterol (mg/dL)					
TC/HC	100.6	23.54	99.95	32.92	0.944
	3.49	0.71	3.39	0.13	0.675
Psychological Parameters					
1. Hamilton Rating Scale for Anxiety	8.66	1.52	1.66	2.88	0.022
2. Multi‐Dimensional Emotional Questionnaire	33.75	7	40.66	4.31	0.011
3. Pittsburgh Sleep Quality Index	5.33	1.52	4.33	2.08	0.001
4. Mini Mental State Exam	27.83	0.28	29.66	0.28	0.044
5. Perceived Stress Scale	14	6.082	6	5.29	0.016
6. WHO QoL BREF	67.83	5.96	82.83	13.65	0.011
7. Oxford Happiness Scale	4.39	0.20	5.29	0.76	0.003

Abbreviations: BDNF, Brain Derived Neurotrophic Factor; HDL, High‐density Lipoprotein; LDL, Low‐density Lipoprotein; SD, Standard Deviation; TC/HC, Total Cholesterol/High‐density Lipoprotein; WHO QoL BREF, World Health Organization Quality of Life Brief Version.

## 4 DISCUSSION

Among the 22 concepts put in the module 19 had CVR of 1, ACP of 100%, and CVI of 1. Two concepts were deleted, and one was modified. According to the feedback of the patients the overall experience of the session was soothing and calming to them which might have helped in bringing down the anxiety and stress level of patients which is the major etiological factor for hypertension. They were able to handle their emotions better especially the negative emotions. The frequency and span of happiness increased and they felt they were able to handle the situations of life better. After the intervention of 4 weeks the level of biochemical parameters BDNF showed significant increase (*p* = 0.008) but Angiotensin II values showed diverse variability, the cortisol levels decreased in six patients among 9 and lipid profile showed very slight improvement. It can be assumed that intervention period was short to see the significant difference in Angiotensin II and lipid profile and the duration of the intervention can be increased from 4 to 12 weeks to see significant difference. In the psychological parameters all the questionnaires showed significant change as decrease in anxiety (*p* = 0.022) and perceived stress (*p* = 0.016), improvement in emotional quotient (*p* = 0.011), and mental state (*p* = 0.044), improvement in quality of life (*p* = 0.011), quality of sleep (*p* = 0.001), and happiness scale (*p* = 0.003).

An alteration in peripheral BDNF concentration by mind body exercise in neurodegenerative disorders and other diseased conditions is reported but the need of more evidence and studies related to effect of BDNF on cognition, brain functions and identify potential difference between mindfulness meditation and mind‐body exercise is felt (You & Ogawa, 2020). The effect of yoga therapy along with meditation on BDNF in neuroplasticity and neuroinflammation by the possible mechanism of having impact on pro‐inflammatory cytokines is established (Estevao, 2022). Meditation being part of the yoga therapy and evidence of upsurge in serum BDNF and decrease in serum cortisol levels by the antidepressant effect is also found (Naveen et al., 2016). A systematic review and meta‐analysis also reports that mindfulness meditation‐based interventions has potential to increase peripheral BDNF levels (Gomutbutra Patama et al., 2020). The findings of the effect of DMM on BDNF and how it affects the health, quality of life, and emotional state of individuals might be used to give early intervention to patients with history of parents having hypertension, working in stressful conditions and emotional imbalance state. Understanding the impact of altered BDNF levels in hypertensive patients with DMM on various factors related to cardiovascular diseases and brain functions might be explored.

The studies done on hypertensive patients are primarily categorized into three groups. First yoga intervention along with meditation/breathing exercises, second analysis of effect of diet, exercise and yoga therapy and third effect of mind body exercise and meditation. In order to analyse the effect of meditation separately was the aim to design the meditation module so that it can gave the real effect of meditation in contrast to combined effect of intervention on patients. The systematic review on mindfulness‐based stress reduction for hypertensive patients for over 30 years also concluded the need of well conducted trials (Conversano et al., 2021). In contrast to studies showing positive effect of meditation and mindfulness, a study done showed less influence of mindfulness and relaxation when used as intervention in treating hypertension and cardiovascular diseases (Zhang et al., 2021). The possibility of getting less effect might be design of the trial or the sample size. Meditation places a clinically significant decrease in elevated blood pressure among patients (Goldstein et al., 2012). When Ambulatory monitoring of blood pressure is done then mindfulness meditation is found as an effective tool in reducing blood pressure especially clinically measured systolic blood pressure (Ponte Márquez et al., 2019). Mindfulness based blood reduction helps in changing behavioral determinants of elevated blood pressure by self‐regulation engagement among patients with hypertension (Loucks et al., 2019). The moderating effect of mindfulness in treatment of somatization symptoms is recognized among female patients with hypertension (Shen et al., 2023). Thus, a structure specifically designed for hypertensive patients and not starting right away with meditation, as all the patients were non‐practitioners of meditation, was kept in mind. The DMM started with mindfulness and then moved to meditation thus not only resolved non‐ specificity but also provided gentle shift of awareness from gross to subtle aspects, self‐ realization and ultimately to meditation by merging self into universal self.

During meditation, sitting in auspicious pose provides a comfortable and stable position of the body to make the mind more and more steady for the process of meditation (Gore, 2017). A person can sit in the desired position for long duration without much distractions. As there are less sensory inputs and perception, the mind develops the ability to concentrate. The mind is tranquilized which is the ultimate aim of meditation. As the spine is erect it prevents compression of abdominal organs and leaves abdominal muscles free for breathing. It facilitates easy movement of the diaphragm. The semi‐conscious state prevents sleeping. Balanced state of body, neck and head stimulates proprioceptors in the spinal muscles to maintain the equilibrium and alternate contraction and relaxation brings stretch reflex mechanism (Gore, 2017). As the focus moves from the external environment to the inner, an inner awareness is felt. As the cardio‐respiratory activity is reduced due to low oxygen demand of the body and reduced production of carbon dioxide on account of negligible muscle activity, it placed less burden on the circulatory system (Gore, 2017). As the knee joints are flexed it restricts the blood flow to the lower extremities thus reducing force to return the venous blood against gravity. The blood flow is directed towards the pelvic and lumbar region enabling nerves plexuses in this region to get richer supply of blood which makes then refreshed and toned. Lower abdominal organs and spine is also tones which gives richer supply of blood to the sacral region which is the place of location of the network of the parasympathetic nerves. These autonomic nerves create a peaceful and pleasant altitude by toning up (Gore, 2017). During DMM practice the patient sits in comfortable posture with back erect and eyes closed to experience relaxation of body muscles, stability of body and calming of the mind due to reduced distractions from the external environment thus bringing changes in heart rate variability, reduction in anxiety and stress, regulation of blood pressure and reduction of factors associated with risk of cardiovascular disorders, which are all the associated benefits of meditation (Ponte Márquez et al., 2019). DMM facilitates meditator to focus and concentrate more and avoid wandering of the mind making it friendly for the practitioner as compared to regular meditation where the meditator meditates without specific directions and autonomously.

Respiratory system also contributes to the state of consciousness, awareness and attention being a semi‐ voluntary action in nature so subtle and rhythmic breathing requires less efforts. Breathing is also related to mind so when we become mindful about our breathing it decreases interference from the external environment and distractions of the mind. From the concept of normal breathing till observations of the thoughts the aim is to bring awareness from external world to the inner environment. During meditation breathing becomes slow, rhythmic and subtle which brings modulations in baroreflex sensitivity used to assess autonomic nervous system functions and regulate blood pressure (Pingali & Hunter, 2023). Even in the DMM practice this phenomenon is observed.

As the heart center is located in the center of the chest region and is associated with emotions like love and compassion, so concentration on it decreases anxiety (Verma & Verma, 2024) which is the causative factor of hypertension. Chanting of seed sound of heart center enables to create subtle vibrations in the region to activate it and release blocked emotions which evokes a sense of empathy towards all and in turn helps in supplying more life force to the area relieving burden on the heart. Subtle life force provides energy to different organs including mind and controls vital life processes in the body as circulation and respiration (Gore, 2017). Breathing system is also linked with nervous system of the body and mind. It is found that slow breathing can reduce blood pressure among hypertensive patients (Herawati et al., 2023) and during meditation breathing becomes subtle and rhythmic enabling control of the nervous system and the mind. During the process the sympathetic arousal is checked and parasympathetic system dominates. As parasympathetic nervous system is associated with regulation of blood pressure so it helps hypertensive patients. Controlling of the mind enables control of different emotions and natural instincts of the mind which brings noticeable relaxation, tranquility, balance and sense of well‐being which is the state of salutogenesis which promotes self‐healing and well‐being. Being mindful induces behavioral changes which promote self‐ regulatory activity, a key factor in controlling chronic disorders (Paley & Johnson, 2023). Neural processes and brain regions that impart benefits to the cognition, emotional regulation, attention and thinking patterns by meditation is found by structural imaging (Marchand, 2014). It is also found that it induces a type of automatic cognition called self‐referential thinking which effects anxiety (Marchand, 2014). As this module also focusses on being mindful and then move to meditation so might be able to alleviate anxiety a causative factor for hypertension. After sitting for a long in the auspicious position, the body becomes free from sensory feedback and conscious effort to maintain the posture. In this position attention to inner sensations and stimulations of the visceral‐ receptors and proprioceptors in the coccygeal, sacral and lumbar region is experienced.

After being mindful about the gross and subtle aspects of body and mind shift to meditation which involves inhibition of mental activities and being aware about self‐consciousness leading to self‐realization (Fasching, 2008). Consciousness awareness makes the person to not to look activities of the mind but making them come to rest. It is not the observance or awareness of anything which leads to non‐introspection and non‐identification with any object. Consciousness is not the state of awareness but the causative force of our existence (Goswami, 2024) which is related to inner existence. As our consciousness exists so does the consciousness of the universe which is the cause for the existence of the universe. The difference between the self and universal consciousness is the vastness and purity. Merging of self and universal consciousness elevates us from the bodily sensations and brings psychosomatic and physiological changes in the body which facilitates enlightenment where we are left with only bliss and pure consciousness. In deep meditation even the consciousness of internal and external world is lost and one looks only into the void (Upanishad, n.d.) but this higher aspect of meditation is not covered in this module of meditation.

Nonconventional interventions like meditation improve the symptoms related to anxiety (Saeed et al., 2019) and the anxiety level decreased among the patients practicing DMM. The result of a systematic review and meta‐analysis shows moderate reduction of psychological stress related to negative dimensions of it (Goyal et al., 2014) which was similar to the decrease in the stress level scores by the intervention of DMM. Daily meditation affects the functioning of prefrontal and hippocampus areas of the brain and helps in emotional regulation by decreasing negative mood scores (Basso et al., 2019), the intervention in this study also showed improvement in the emotional quotient in the patients. They were able to regulate both the positive and negative emotions but the regulation of the negative emotions was better as compared to positive emotions. Meditation improves the mental health is found (Zollars et al., 2019) and in this study also the score of mental state improved after the DMM intervention. Sleep quality improves by the practice of meditation (Rusch et al., 2019) and even DMM there was decrease in the scores of sleep disturbance indicating improvement of the sleep quality. Meditation significantly improves the quality of life (Cavalcante et al., 2023) and DMM also showed improvement in the all the domains of the quality of life physical, psychological, social and environmental along with improvement in the overall quality of life and health scores. Result of this pilot study showed improvement in the happiness state as shown by a study on college students taking meditation course improved well‐being (Crowley et al., 2022).

Practicing meditation effects the endocrine system including renin‐angiotensin‐aldosterone system requires exploration (Pascoe et al., 2020) and even in this study we did not find statistically significant results in the decrease in the levels of angiotensin II among the patients after the intervention. Angiotensin II values showed diverse variability and further study with longer duration of intervention is felt to see the significant difference. Mindfulness intervention modulates the cortisol levels in response to stress (Fan et al., 2024) and DMM showed decrease in cortisol levels. Meditation as om chanting improves lipid profile in the hypertensive patients (Anjana et al., 2022) but very slight improvement was found in this study in the lipid profile due to short duration of the intervention for a month and necessity to increase the duration of intervention for 3 months is felt to see the significant results.

During the process of DMM practice various aspects which were handled sub‐consciously were negative and unwanted emotions, cause of stress and anxiety due to attachment and expectations, melancholy state of mind, lack of concentration and illusion about being. The effect of DMM will be analyzed on larger sample size and randomized controlled trials will be performed in future to provide more evidence related to effect of DMM.

## 5 CONCLUSION

The DMM can be given to PHP as it was found to be valid by the experts and feasible to the patients and was also found to be efficient in increasing BDNF levels, reducing anxiety and stress, improve emotional quotient, mental state, quality of life and happiness among hypertensive patients. It is also influencing the etiological factors of hypertension and is a preventive measure for the development of complications of hypertension.

### 5.1 Limitations

This study was done on a small sample size to check the feasibility and efficacy. It can be done with a bigger sample size with increased duration of intervention to 3 months to the patients in the future to increase the impact and analyze benefit on the PHP. Moreover, a control group can also be introduced in the future study to analyze the statistical difference between the groups. As the focus of this study was to design a meditation module and check its feasibility and efficacy with respect to BDNF, anxiety, stress, emotional quotient, mental state, quality of life and happiness, parameters such as blood pressure, pulse rate and respiratory rate were not recorded post intervention and in the future studies all these parameters will also be recorded.

## AUTHOR CONTRIBUTIONS

Conceptualization: Kapila Goswami Sharma, Dr Manjula SD, Dr MG Ramesh Babu; Methodology: Kapila Goswami Sharma, Dr Manjula SD, Dr MG Ramesh Babu, Dr Ujjal Bose; Patient Recruitment: Dr M Mukhyaprana Prabhu, Formal analysis and investigation: Dr Shobha U Kamath, Dr Ujjal Bose; Writing Draft and Editing: Kapila Goswami Sharma, Dr Manjula SD; Supervision: Dr Manjula SD, Dr Vasanthalaxmi K.

## FUNDING INFORMATION

No funding information provided.

## CONFLICT OF INTEREST STATEMENT

The authors have no competing financial and non‐financial interests directly or indirectly related to this work.

## ETHIC STATEMENTS

This study is approved by Kasturba Medical College and Kasturba Hospital Institutional Ethics committee (IEC 1‐154) and registered prospectively in the Clinical Trials Registry‐ India (CTRI/2022/09/045421) on 12/09/2022. The study was performed in accordance with the ethical standards as laid down in the 1964 Declaration of Helsinki. Permission from the Head of the Institution and Medical Superintendent to recruit the patients was taken prior to recruitment of the patients and from the authors to use the questionnaires. The written consent on the Informed consent form was taken from all the participants in the study prior to their involvement in the study.

## DISCLAIMER

This is a confidential document.

## Data Availability

The datasets used and analyzed in the current study are available from the corresponding author upon reasonable request.
